# How Is the Neural Response to the Design of Experience Goods Related to Personalized Preference? An Implicit View

**DOI:** 10.3389/fnins.2018.00760

**Published:** 2018-10-26

**Authors:** Yongbin Ma, Jia Jin, Wenjun Yu, Wuke Zhang, Zhijiang Xu, Qingguo Ma

**Affiliations:** ^1^Business School, Ningbo University, Ningbo, China; ^2^Academy of Neuroeconomics and Neuromanagement, Ningbo University, Ningbo, China; ^3^Modem Management Research Centre, Ningbo University, Ningbo, China; ^4^College of Information Engineering, Zhejiang University of Technology, Hangzhou, China; ^5^Institute of Neuromanagement Science, Zhejiang University of Technology, Hangzhou, China

**Keywords:** event-related potentials, experience goods, late positive potential, P200, personalized product designs

## Abstract

Understanding the process by which consumers evaluate the designs of experience goods is critical for firms designing and delivering experience products. As the implicit process involved in this evaluation, and given the possible social desirability bias inherent to traditional methods of product design evaluation in certain conditions, neuroscientific methods are preferred to gain insight into the neural basis of consumers’ evaluation of experience good designs. We here used event-related potentials (ERPs) and a revised go/no-go paradigm to investigate consumers’ neural responses to experience good designs. Personalized product designs and neutral landscape pictures were randomly presented to 20 student participants; they were asked to view these product designs without making any decisions. The paired *t*-test and repeated-measures analysis of correlation showed that the P200 and late positive potential (LPP) elicited by the most-preferred experience good designs were significantly higher than that elicited by least-preferred designs, and the two ERP components were positively correlated with the personalized rating scores. Thus, P200 and LPP might be the early and late indices of consumers’ evaluation of experience good designs, respectively, and may facilitate an understanding of the temporal course of this evaluation. Furthermore, these two ERP components can be used to identify consumers’ preferences toward experience good designs. In addition, given the use of personalized experimental stimuli, these findings may help to explain why customized products are preferred by consumers.

## Introduction

Design is an inherent component of all products, and has a lasting effect on consumers’ loyalty to brands and their purchasing decisions (Reimann et al., 2010; Homburg et al., 2015). Experience goods, such as clothes, wines, and cosmetics, are products whose utility cannot be ascertained before purchase, because of the lack of full information (Nelson, 1970, 1974). Product design is especially important for such goods, because product design is an important source of utility of experience goods. For example, besides the functional dimension, the esthetic and symbolic dimension of T-shirts are also important parts of T-shirt designs (Homburg et al., 2015), and they are also the key factors influencing consumers’ experience evaluation of T-shirts. More important, product design is the main source of uncertainty in consumers’ evaluation of experience goods. Compared to search attributes of experience products, such as material and price, consumers’ evaluation of experience attributes of these goods, such as product designs, is more subjective (Huang et al., 2009), and their needs for experience attributes are more personalized (Bloch et al., 2003). Therefore, it is difficult for enterprises to understand consumers’ demand for experiential good designs effectively, and for consumers to use the information provided by firms to judge the fit between their needs and experience good designs, which will increase the uncertainty of consumers’ evaluation of experiential products. In practice, some T-shirt, mobile phone case, and cake firms have even given up on understanding of the consumers’ needs for experience product designs, and have outsourced the product design task to consumers through personalized customization (Schreier, 2006). The basic premise of personalized customization is to let consumers choose the design elements that they like, while the firms complete the production and distribution. Therefore, exploring consumers’ process of evaluating experience product designs can shed light on the consumer needs for experience products and provide important information to enterprises for designing and delivering experience goods.

The evaluation of product designs is an esthetic evaluation process, which has been found to be associated with consumers’ cognitive and emotional responses (Silvia and Warburton, 2006), and is influenced by the existing knowledge and experience of consumers. This makes the consumers’ evaluation of product designs more subjective and personal. Therefore, relatively objective measures are needed to capture the process and characteristics of product design evaluation (Luo et al., 2008; Pearce et al., 2016). Additionally, the implicit information processing involved in this esthetic evaluation process of product designs makes the traditional questionnaire method less effective in this study (Wang and Tseng, 2015). For example, questionnaires can be used to elicit consumers’ explicit preference for experience product designs, but it cannot explain the implicit reasons for this preference. In addition, for products that satisfy consumers’ social communication and status-seeking need (Heine and Phan, 2011), such as luxury goods, and for participants who participate in product preference evaluation in order to obtain monetary rewards (Davidson et al., 2002), directly eliciting consumers’ response to product designs using questionnaire may cause social desirability bias.

The event-related potential (ERP) technique can directly measure individuals’ perceptual and cognitive processes in response to stimuli with high temporal resolution (Luck et al., 2000), and can help to record the activities that involve social-desirability biases or are otherwise difficult to report (Cerf et al., 2015). It can also help to discover physiological factors that influence individual behavior and preferences and explore the “common scale” that allows comparison of heterogeneous and individualized behavior (Levy and Glimcher, 2012; Camerer and Yoon, 2015). Studies have explored the neural processes and brain regions involved in the evaluation of esthetic objectives by means of EPRs, for example, arts (Augustin et al., 2011) and faces (Chatterjee et al., 2009). For product designs, in addition to the esthetic aspect, such as arts and music, the symbolic aspect is also important and is significantly related to consumer behavior (Homburg et al., 2015).

Previous studies have also adopted the ERP technology to study consumers’ preference using product images and have indicated that some ERP components can effectively predict consumer preferences and choices (Junghöfer et al., 2010; Pozharliev et al., 2015; Telpaz et al., 2015). Nevertheless, these studies did not considered differences between different types of products, except for the study by Pozharliev et al. (2015), which explicitly focused on luxury goods. In fact, consumers consider different factors when purchasing different types of products. For example, for search goods, the objective attributes (e.g., price and functions) are influential in the decision-making process (Huang et al., 2009). The EEG signals collected when consumers view the product images (subjective attributes) of search goods cannot capture the key information of consumers’ decision-making process, and the predictive relationship of EEG signals and product preferences is also not accurate. For experience goods, the subjective attributes (e.g., product designs) are important (Hoch and Ha, 1986). EEG signals collected when consumers view the product images can capture the process of consumers’ evaluation of product (designs) more accurately. In addition, in these studies, all the subjects were assigned to view the same product design images, and the subjective and penalized demand of consumers for product designs was not appropriately considered. For experience goods, for which subjective attributes are important (Hoch and Ha, 1986), EEG signals collected when viewing the same product design pictures as previous studies may not capture the individualized differences in consumers’ evaluation of product designs. Thus, by using personalized experimental stimuli and ERP technology, this study focused on the process of evaluation of experience product designs.

Product design evaluation is correlated with esthetic evaluation (Bloch, 1995; Silvia and Warburton, 2006). Previous studies have posited that esthetic evaluation consists of two distinct stages: early impression formation and a subsequent evaluative categorization stage (Celaconde et al., 2013). During the early impression formation phase, individuals devote more attentional resources to exploring stimuli. The subconscious processes and visual properties of the stimuli are involved in this stage (Celaconde et al., 2004). P200 is a positive-going waveform with a peak latency at about 200 ms after the onset of stimuli, and is related to early automatic and selective attention (Olofsson et al., 2008). P200 can be elicited by affective stimuli, reflecting the initial sensory encoding of emotionally significant stimuli, and the onset of a persistent positive shift of the ERP waveform (Olofsson et al., 2008). As individuals pay more visual attention to product designs that they find attractive (Coates, 2003), we speculated that a greater positive-going amplitude would be observed in P200 for the most-preferred product designs of experience goods than for the least-preferred designs.

In the evaluative categorization stage, more conscious and cognitive appraisals are triggered, which result in a more enduring esthetic judgment and emotional response (Kumar and Garg, 2010; Celaconde et al., 2013). The late positive potential (LPP) is a long-lasting ERP component peaking at around 500–700 ms after the onset of stimuli. An enhanced LPP amplitude represents a reliable, replicable, and time-specific emotional response to stimuli (Hajcak et al., 2006). The LPP amplitude is positively correlated with arousal level, which implies that pictures causing high emotional arousal (pleasant or unpleasant, rather than neutral) elicit augmented LPP (Schupp et al., 2000). The LPP also indicates the sustained enhanced attention allocation and motivational significance of emotional visual stimuli during affective perceptual processing (Bradley et al., 2003). As most-preferred product designs of experience goods will elicit more emotional arousal than less-preferred designs, and individuals reliably allocate more attention to the former (Coates, 2003), we speculated that greater positive-going amplitude of LPP would be observed for the most-preferred experience goods designs than that for the least-preferred designs. Moreover, according to neuropsychological models of attention, P200 and LPP are correlated and jointly reflect different attentional processes for the same visual stimulus (Pozharliev et al., 2015). Therefore, we speculated that the two ERP components would both be evoked during the product design evaluation process of experience goods.

In this study, we analyzed the neural activities related to the design evaluation of experience goods. We speculated that more positive-going ERP amplitude would be observed for the two ERP components, P200 and LPP, for the most-preferred experience good designs than that for the least-preferred designs. To investigate the personal and subjective nature of product design evaluation, we used personalized product designs rather than the same stimulus for all the participants in the experiment, since personalized stimuli may reflect individuals’ preferences for product designs more accurately (Pearce et al., 2016).

## Materials and Methods

### Study Participants

The participants were 20 students recruited from Ningbo University, aged 18–26 years of age (mean age = 24 years, SD = 2.247; 53% women). All the participants reported normal or corrected-to-normal vision and had no history of neurological or psychiatric illness. The participants were paid ¥40 as compensation for participation in the study. Four of the participants were removed from the study due to excessive ERP artifacts. This study was approved by the local institutional ethics committee of the Academy of Neuroeconomics and Neuromanagement at Ningbo University. All participants provided written informed consent before the experiment started.

### Study Materials

Considering the familiarities of the participants and the needs for personalized experimental stimuli, we selected the T-shirts as experimental stimuli. Using the three-dimensional scale reported by Weathers et al. (2007), we measured the perceived product type of T-shirts of 30 students (recruited from the same university as those for the main experiment), the average score of the three dimensions was 4.910 (1 = pure search goods, 7 = pure experience goods), which indicate that T-shirts were more likely to be perceived as experience rather than search goods.

The current common practice of implementing “personalized T-shirt design” is as follows: first, firms post design elements on their website; then, consumers choose and combine design elements by themselves; finally, T-shirts are manufactured and derived according to the consumers’ preference. As this is time-consuming and is not easy to implement in the laboratory, we asked the research assistants to select the images of the T-shirt designs of five major T-shirt brands from their official websites on Tmall.com (choosing the new and personalized designs). We also used the Baidu search engine to select 40 neutral landscape images for implicit tasks. All the pictures were processed to have a white background, a resolution of 360 × 270 pixels, and were in pdf format. The size of these pictures was 360 mm × 360 mm. There were no explicit brand names, logo, or other explicit signals on the product design pictures.

### Experimental Procedures and EEG Recording

**FIGURE 1:**
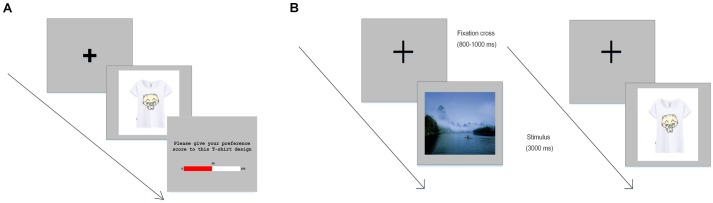
Example trials of the experiment. **(A)** T-shirt preference rating task. During each trial, after a fixation cross appeared, participants were presented with a picture of a T-shirt, and then they were instructed to give their preference scores for the T-shirt. **(B)** Landscape picture and product design presentation in the ERP task. In each trial, after a fixation cross appeared, landscape pictures and product designs were presented randomly. Participants were instructed to view these pictures without making any decisions regarding the product designs and to press a key for landscape pictures.

The experiment was divided into two stages (Figure 1). In the first stage, participants were told that these T-shirt designs were randomly combined using the design elements of T-shirt customization companies on Tmall.com, and they were asked to indicate their preference for these T-shirt design pictures. Their preference was measured using a 0–100 horizontal preference scale (100 = completely prefer the design pictures). In order to increase the accuracy of subjective measurement, participants were told that “there are no right or wrong answers in the evaluation of the T-shirt designs; please give your true preference. Your evaluation will only be used for this study, and we will keep your answers strictly confidential.” We did not record the name and other personal information of these participants. The survey was conducted in an independent behavioral laboratory and there was no interference throughout the process.

After scoring, participants were invited to attend the second stage of the experiment, which was conducted in a dimly lit and electrically shielded EEG laboratory. The design of this experiment followed a revised go/no-go paradigm, in which the participants were instructed to “go” (press a key) when a landscape picture was presented, and to “no-go” (refrain from pressing a key) when a T-shirt picture was presented. The ERP experiment consisted of three blocks, and each block contained 40 trials. For each participant, there were two groups of 40 personalized T-shirt pictures (most-preferred and least-preferred product designs), and one group of 40 landscape pictures. The two groups of T-shirt pictures were classified based on the self-rating scores allotted to these pictures in the first stage. The 40 landscape pictures were discarded at the data analysis stage. All the pictures were presented in a randomized order. Participants were instructed to watch these pictures without making any overt responses, and to minimize head and body movements during this stage.

The pictures were presented on a 19-in monitor (1280 × 1024 pixels, 60 Hz), which was connected to a 2 GHz Pentium computer. E-prime 2.0 (Psychology Software tools, Pittsburgh, PA, United States) was used for stimulus presentation and data collection. The pictures were positioned at the center of the screen and viewed from a distance of 100 cm, at a visual angle of 6.27–6.271°. The background of the screen was gray (RGB: 128, 128, 128).

Event-related potential data were recorded using 64 Ag/AgCl electrodes mounted on an elastic cap with a Neuroscan Synamp2 Amplifier (Scan 4.3.1, Neurosoft Labs, Inc.). A forehead location was used for grounding, and the reference was the left mastoid. Using left and right mastoid references, data were transferred to the average offline. Vertical electrooculograms were recorded using a pair of electrodes placed above and below the right eye, while horizontal electrooculograms were recorded using another pair of electrodes placed on the right side of the right eye and left side of the left eye. Both vertical and horizontal pairs of electrodes were placed 10 mm from the eyes. Electrooculogram artifacts were corrected offline. The experiment started with the impedances of the electrodes less than 5 kΩ.

Epochs were made beginning 200 ms before stimulus onset and continuing for 800 ms after the onset. The EEG was aligned to a 200-ms baseline, and error-of-commission artifacts were corrected using the method proposed by (Semlitsch et al., 1986). Trials with bursts of electromyography activity, peak-to-peak deflection exceeding ± 100 μV, and amplifier clipping were excluded. The averaged ERPs were digitally filtered using a low-pass filter at 30 Hz (24 dB/octave). The EEG recordings for every participant were averaged separately over each recording site for each of the most- and least-preferred product design conditions. The data were further analyzed for the two experimental conditions.

### Statistical Analysis

Previous studies have indicated that the modulation of the P200 amplitude to emotional visual stimuli is most pronounced in the posterior scalp areas (Schupp et al., 2004; Pozharliev et al., 2015), and that the LPP is most pronounced in the superior–posterior scalp areas (Sabatinelli et al., 2006; Pozharliev et al., 2015). These two components, elicited by the emotional visual stimuli, are usually reported in both the left and right hemispheres (Dolcos and Cabeza, 2002). Based on these findings, for P200, we analyzed signals from the following nines electrodes: C1, CZ, C2, CP1, CPZ, CP2, P1, PZ, and P2. As the nine electrode sites in the posterior area had a similar pattern of results, the data from the region were pooled to obtain a representative value. For the LPP, we focused on the following six electrodes: C1, CZ, C2, CP1, CPZ, and CP2, the data from all the six electrode sites in the central and parietal scalp areas were also pooled for the same reason as for P200.

Based on previous studies and visual inspection of grand averages of waveforms (Pozharliev et al., 2015), the time windows chosen for P200 and the LPP were 160–210 and 500–700 ms, respectively. For each participant, we examined the P200 and LPP amplitude in the most- and least-preferred conditions and matched these amplitudes with the average preference rating scores for the personalized product designs under both conditions. We examined whether there were significant differences in the ERP amplitude for different preference conditions, and how the ERP components induced by product designs were correlated to the corresponding personalized preference rating scores. As product designs that were presented to the participants were nested within the subjects, the pairwise *t*-test and repeated measures analysis of correlation were used.

## Results

The average scores for T-shirt designs of the five brands were 52.71, 51.66, 52.24, 53.23, and 50.47, and repeated measures ANOVA revealed that there was no significant difference between these brands [*F*(4,2964) = 2.279, *p* > 0.05). In addition, we found that the scores of the same T-shirt design were significantly different (the SD of the mean scores was 16.858), which indicated the importance of individualized experimental stimuli. Paired *t*-tests showed that the difference between consumers’ average preference for the most- and least-preferred experience good designs was significant [(M most-preferred = 68.75, M least-preferred = 35.680), *t*(15) = 13.369, *p* < 0.01].

**FIGURE 2:**
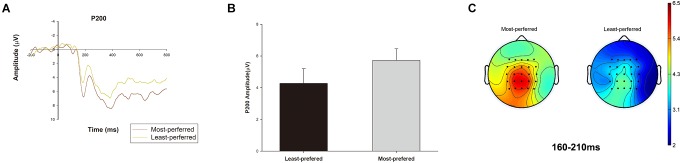
P200 condition effect. P200 waveforms were averaged from the 16 subjects, and we pooled the data from C1, CZ, C2, CP1, CPZ, CP2, P1, PZ, and P2 electrodes. The time window was 160–210 ms for P200. **(A)** The ERP grand-average waveforms of P200 component in the most-preferred and the least-preferred product design conditions. **(B)** The bar chart reflecting mean amplitude of P200 in the most-preferred and the least-preferred product design conditions. Error bars indicate standard error. **(C)** Topographic maps of the most-preferred and the least-preferred product design conditions for P200 amplitude.

**FIGURE 3:**
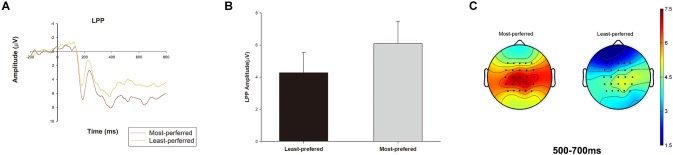
The LPP condition effect. The LPP waveforms were averaged from the 16 subjects, and we pooled the data from C1, CZ, C2, CP1, CPZ, and CP2 electrodes. The time window was 500–700 ms for the LPP. **(A)** The ERP grand-average waveforms of the LPP component in the most-preferred and the least-preferred product design conditions. **(B)** The bar chart reflecting mean amplitude of P200 in the most-preferred and the least-preferred product design conditions. Error bars indicate standard error. **(C)** Topographic maps of the most-preferred and the least-preferred product design conditions for the LPP amplitude.

A pairwise *t*-test indicated that the P200 amplitude was larger for the most-preferred than for the least-preferred experience good designs [*t*(15) = 3.331, *p* < 0.01, M most-preferred = 5.717, M least-preferred = 4.273; 95% confidence interval [CI]: [0.520 2.367], Figure 2]. For the LPP, a paired *t*-test also indicated a significant difference between the most- and least-preferred experience good designs [*t*(15) = 2.372, *p* < 0.05, M most-preferred = 6.089, M least-preferred = 4.268; 95% CI: [0.185, 3.456], Figure 3].

Repeated measures analysis of correlations indicated that the P200 amplitude and average preference rating scores for the experience good designs were significantly positively correlated (*r* = 0.553, *p* < 0.05, 95% CI = [0.056 0.830]). The LPP amplitude and average preference rating scores for the experience good designs were also significantly positively correlated (*r* = 0.606, *p* < 0.01, 95% CI = [0.136, 0.853]). These results indicated a positive correlation between the P200 and LPP amplitude and the consumer preference rating scores, which implies that the higher the P200 and the LPP amplitudes were, the higher were the personal preference ratings for the product designs of experience goods.

## Discussion

This study investigated consumers’ neural responses to the most-preferred and least-preferred experience good designs. Using personalized T-shirt pictures, we found that, compared to the least-preferred product designs of experience goods, the P200 and LPP amplitudes were consistently enhanced for the most-preferred designs. Furthermore, the mean amplitudes of P200 and the LPP were significantly positively correlated with the consumers’ average preference ratings.

From a theoretical standpoint, compared to the study of Telpaz et al. (2015), which only demonstrated a positive relationship between an increased early ERP component (N200) and consumers’ future choice (Telpaz et al., 2015), we demonstrated that, compared to viewing pictures of the least-preferred product designs, viewing pictures of the most-preferred experience good designs elicited larger amplitudes in early and late (P200 and LPP) ERP components. This may be because we focused on experience rather than on search goods. Previous studies have shown that the late component, LPP, was associated with the arousal of emotional stimuli. For highly arousing emotional stimuli (whether positive or negative), the amplitude of the LPP was steadily increased as compared to neutral stimuli (Schupp et al., 2000, 2004). Minnix et al. (2013) have also showed that images with high-emotional arousal induce higher LPP amplitude than images with low-emotional arousal, while the LPP amplitude did not differ between images without emotional arousal (neutral objective images). In addition, other studies have also showed that the esthetic experience evaluation process includes two different stages: early impression formation and post-evaluation classification (Celaconde et al., 2013). P200 and the LPP can represent the early attention arousal and the late emotional cognition assessment, respectively (Parasuraman and Beatty, 1980). Based on these studies, it is believed that, for product designs with high emotional arousal, later ERP components will be enhanced. In the process of evaluating search good designs, consumers focus on search attributes (e.g., functions, shape, and dimensions). The emotional arousal level induced by these search attributes was lower than by the experience attributes. Therefore, there was no difference between the LPP component in the preferred and unreferred product conditions. Experience good designs are related to symbolic and esthetical dimensions of product design, which are related to emotional experience and arousal (Homburg et al., 2015). This explains why the amplitude of early and late ERP components were both enhanced for experience goods.

For the early components, this study indicated that, compared to viewing the pictures of the least-preferred experience good designs, viewing pictures of the most-preferred designs elicited a larger P200, rather than N200, amplitude. This result indicates the role of early attention in the evaluation of experience good designs. Previous studies have indicated that early ERP components (such as P200) reflect the perceptual processing of visual stimuli (Parasuraman and Beatty, 1980); a larger degree of visual stimulation (such as the most-preferred product designs) evoked more attentional arousal. However, consumers who purchase search goods will more likely to consider the objective attributes (e.g., price and function) and economic rewards of these attributes (Huang et al., 2009). As search goods (attributes) that are not preferred by consumers will result in lower reward outcomes than they anticipated (Baker and Holroyd, 2011), the N200 component in the early stage will be enhanced to represent this mismatch (Pozharliev et al., 2015). Therefore, ERP amplitudes enhancement differed for search and experience goods. In addition, previous studies have indicated that P200 not only reflects the initial response to esthetic stimuli, but is also related to the subsequent approach and withdrawal behavior (Schapkin et al., 2000). For example, early neural activities (such as P200) were related to later subjective behavior during environmental risk evaluation (Qin and Han, 2009). These results support the conclusion that the P200 component was positively related to the subjective preference evaluation in our study.

As for the LPP, we found a more enhanced LPP amplitude at around 600 ms for the most-preferred product designs than for the least-preferred designs after exposure to experience good designs. We also found that the LPP amplitudes were positively correlated to consumers’ personal preference scores. Based on a previous study (Bradley et al., 2007), we can infer that the most-preferred product designs of experience goods evoked greater emotional arousal than the least-preferred designs. Besides the esthetic dimension, the enhancement of the LPP component in this paper may also be related to the symbolic dimension of experiential product designs, because the symbolic meaning of the product design is also related to affective product attributes (Homburg et al., 2015). The increased LPP amplitude also reflected motivated attentional processing and motivational intensity (Ferrari et al., 2011), implying enhanced allocation of relevant resources to promoting and speeding up the processes leading to a suitable response to the stimuli (Lang et al., 1997). Previous studies have shown that enhanced LPP reflects the increased positive emotional motivation for a preferred brand and represents a positive buying willingness for the brand (Bosshard et al., 2016). Therefore, in line with previous studies, we observed that LPP was related to behavioral responses to product designs of experience goods.

At a practical level, this study established that the two ERP components, P200 and the LPP, were positively correlated with the evaluation of experience good designs, implying that ERPs can be used to predict consumers’ personal preferences for product designs of experience goods. In addition, as this study used the personalized experimental stimuli, the results can also help to understand the consumer’s evaluation of personalized product designs. This study further indicated that ERPs can be used to predict consumers’ preference for experience good designs, without them making actual decisions. EEG signals are of great use when rating data are not available or are limited and may help to reduce responses and inference biases in a practical context.

Compared to previous studies, we used subjective rather than objective ratings (for example, sales or other measures) to assess consumers’ preferences for experience good designs. Such subjective measures have been recommended to capture the personal and subjective nature of esthetic evaluation (Chatterjee et al., 2009; Conway and Rehding, 2013; Pearce et al., 2016). We assigned experimental stimuli based on the individual preferences of the participants. We found that participants’ preferences for the same experience product design varied greatly. Therefore, if we had not used personalized materials, we could not have observed differences in the EEGs during the process of evaluating experience product designs. In addition, by using passive viewing rather than direct assessment of experience good designs, our results indicated that the ERP components evoked by experience good designs were related to consumers’ preferences. These results are consistent with those of previous studies, which have indicated that even without paying explicit attention to pictures (not required to provide assessment), participants can still exhibit a neural response to the pictures (Chatterjee et al., 2009; Pozharliev et al., 2015).

This study had some limitations. First, although this study focused on experiential products, we only used T-shirts as experimental stimuli. Whether the results of this study can be extended to other experience goods requires further attention. Second, in order to capture consumers’ subjective and personalized characteristics of product design evaluation, we used the subjective preference scores of each participant to classify experimental stimuli into two groups. Although we used various methods to maximize the accuracy of subjective measurement, a social desirability bias may still remain. Third, we did not directly compare the ERP response between experience and search goods. Although we focused on experiential products, the concept of experience products was proposed relative to search goods (Nelson, 1970, 1974). It may be better to compare the differences in ERP responses between the two types of product designs directly.

## Conclusion

This study explored individuals’ neural responses to experience good designs and their relation to personal preferences. Using personalized T-shirt designs as stimuli, the results indicated that two ERP components, P200 and the LPP, were both enhanced in response to the most-preferred product designs of experience goods as compared to the least-preferred designs, when participants simply viewed the product designs without making actual decisions. Both of the ERP components were positively correlated with the consumers’ preference scores for these experience good designs. The results indicated that ERP signals may provide important information regarding consumer preferences for experience good designs and can shed light on why consumers like customized products.

## Ethics Statement

This study was carried out in accordance with the recommendations of the Ethics Committee of the Academy of Neuroeconomics and Neuromanagement at Ningbo University with written informed consent from all subjects. All subjects gave written informed consent in accordance with the Declaration of Helsinki. The protocol was approved by the Ethics Committee of the Academy of Neuroeconomics and Neuromanagement at Ningbo University.

## Author Contributions

YM made substantial contributions and participated in all aspects of the paper, conducted the experiment, analyzed the data, and wrote the manuscript. JJ and WY made substantial contributions to the work and participated in all aspects of the paper. WZ and ZX participated in the data acquisition and data interpretation stage. QM oversaw the study and managed every part of research. All authors read and approved the final manuscript.

## Conflict of Interest Statement

The authors declare that the research was conducted in the absence of any commercial or financial relationships that could be construed as a potential conflict of interest.
